# The structures of a naturally empty cowpea mosaic virus particle and its genome-containing counterpart by cryo-electron microscopy

**DOI:** 10.1038/s41598-017-00533-w

**Published:** 2017-04-03

**Authors:** Emma L. Hesketh, Yulia Meshcheriakova, Rebecca F. Thompson, George P. Lomonossoff, Neil A. Ranson

**Affiliations:** 1 Astbury Centre for Structural Molecular Biology, University of Leeds, Leeds, LS2 9JT UK; 2 Department of Biological Chemistry, John Innes Centre, Norwich Research Park, Colney, Norwich NR4 7UH UK

## Abstract

Cowpea mosaic virus (CPMV) is a picorna-like plant virus. As well as an intrinsic interest in CPMV as a plant pathogen, CPMV is of major interest in biotechnology applications such as nanotechnology. Here, we report high resolution cryo electron microscopy (cryo-EM) maps of wild type CPMV containing RNA-2, and of naturally-formed empty CPMV capsids. The resolution of these structures is sufficient to visualise large amino acids. We have refined an atomic model for each map and identified an essential amino acid involved in genome encapsidation. This work has furthered our knowledge of *Picornavirales* genome encapsidation and will assist further work in the development of CPMV as a biotechnological tool.

## Introduction

Cowpea mosaic virus (CPMV) is a plant-infecting member of the order *Picornavirales*, with a relatively simple, non-enveloped capsid that has been extensively studied. The *Picornavirales*, which contains >8 families and hundreds of distinct viruses, includes members that infect vertebrates (e.g. foot and mouth disease virus and poliovirus), insects (e.g. acute bee paralysis virus) and plants (e.g. bean pod mottle virus (BPMV), rice tungro spherical virus).

**Figure 1:**
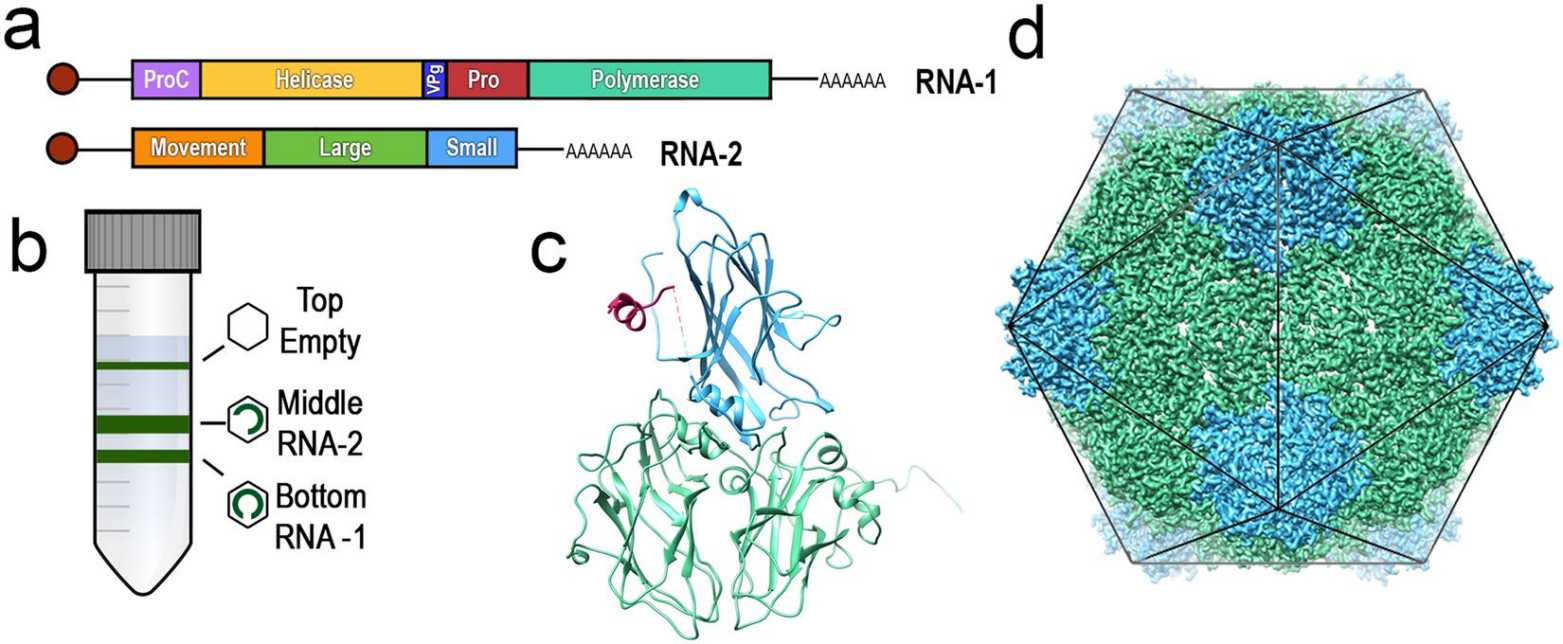
An introduction to Cowpea mosaic virus (CPMV). (**a**) CPMVs single stranded bipartite RNA genome. RNA-1 is ~6 kb in length and encodes viral proteins required for replication. RNA-2 is ~3.5 kb in length and encodes the structural coat proteins and the movement protein required for moving CPMV virions from cell to cell. (**b**) Gradient centrifugation of wild-type CPMV permits separation into three components. Empty CPMV particles sediment at the top (CPMV-T), CPMV containing RNA-2 sediments in the middle (CPMV-M) and RNA-1 containing CPMV particles sediment at the bottom of a density gradient (CPMV-B). (**c**) An asymmetric unit of CPMV empty virus-like particle (eVLP), (PDB 5a33). The large coat protein subunit (L subunit, green) and the small coat protein subunit (S subunit, blue). The C terminal extension, only visualised in a eVLP is coloured pink. (**d**) The icosahedral organisation of CPMV using the EM derived map of eVLP (EMD-3014). Each of the 60 asymmetric units comprises one copy of the L subunit and the S subunit (coloured as in 1C). A view down the two-fold axis is shown.

CPMV is the type member of the *Comoviridae* subfamily and, in common with all members of the *Picornavirales*, has a positive-sense, single-stranded RNA genome. For CPMV, the genome is bipartite, with RNA-1 (6 kb) and RNA-2 (3.5 kb) being separately encapsidated (Fig. 1a). CPMV has an icosahedral capsid structure, which is ~30 nm in diameter and is formed from 60 copies each of a Large (L) and Small (S) coat protein. These two coat proteins are processed from a single RNA-2-encoded precursor polyprotein (VP60) by the action of the 24 K viral proteinase which is encoded by RNA-1. Thus capsid assembly, as well as viral infection, is dependent on the presence of both genomic segments in an infected plant cell. Particles containing the two different genomic RNAs can be readily separated using density gradient ultracentrifugation (Fig. 1b), with particles containing the larger RNA-1 sedimenting more quickly than those containing the smaller RNA-2, whilst a relatively small amount of empty (RNA-free) particles sediments slowest. Thus particles containing RNA-1 are the ‘bottom’ (CPMV-B) fraction, RNA-2 are ‘middle’ (CPMV-M) and CPMV particles that have not packaged a genome are the ‘top’ (CPMV-T) fraction.

The structure of the CPMV capsid is well understood. Crystallographic structures are available for three Comoviruses: CPMV, BPMV and red clover mottle virus^1–5^. Together, the L and S subunits comprise three β-barrel domains (two from L, one from S; Fig. 1c) each of which contains a ‘jelly roll’ topology. The ‘jelly roll’ is common in many virus structures and contains two twisted anti-parallel β sheets, each of which contains four β strands (Fig. 1c). The C terminal 24 amino acids of the S subunit are essential for viral assembly and genome encapsidation^6^; however, these amino acids are cleaved during the normal maturation of the virus^7^ and so are missing from all Comovirus X-ray structures, with the last amino acid observed in the S subunit of CPMV being Lys189^1^. These three jelly-roll domains thus correspond to the three quasi-equivalent conformers of a *T* = 3 icosahedral lattice, and Comovirus capsids therefore adopt a *p*T = 3 quasi symmetry (Fig. 1d). The particle has pronounced turrets formed from the S subunit at the particle fivefold axes. Indeed, the penton of a set of coat protein subunits appears to be the basic building block for all *Picornavirales* capsids: a penton of L & S for CPMV or a penton of VP1/2/3/4 for e.g. poliovirus.

In addition to this wealth of X-ray evidence, recently we have determined two high resolution cryo electron microscopy (cryo-EM) structures for CPMV, with a 3.4 Å structure of the RNA-1 containing bottom fraction (CPMV-B) and a 3.0 Å structure of a recombinant empty virus-like particle (eVLP)^8^. These EM structures are noteworthy for two main reasons. Firstly, whilst the structure of the CPMV capsid is well understood, the organisation of encapsidated genomic RNA within the infectious virus is not. Only for BPMV particles containing RNA-2 can any ordered RNA structure be observed, with ordered ribonucleotides being visible near the particle three-fold axes forming a trefoil shape^4^. Owing to icosahedral averaging it was impossible to deduce the RNA sequence, but the base composition was not random, and it was suggested that these sequences might be critical determinants for assembly or stability of capsids. In the EM structure of CPMV-B, significant extra density corresponding to genomic RNA was observed, albeit at lower resolution (5–10 Å). The trefoil-shaped RNA density was not seen, but rather a dodecahedral cage of RNA density was observed, with discrete bridges of density to the protein capsid that implicated several amino acid residues in genome binding.

The second major point of interest in the previous cryo-EM structures of CPMV, was the presence of additional density representing part of the 24 amino acid, C terminal extension to the S subunit in the eVLP structure. Recombinant eVLPs were produced by transient expression of VP60 and the 24 K protease^9^. This results in eVLPs composed of L and S subunits which have an identical sequence to the three forms of WT CPMV (T/M/B). However, the eVLP retains the C terminal segment better than WT CPMV particles that contain RNA (CPMV-B/M), suggesting an allosteric effect of genomic RNA in accelerating polypeptide backbone cleavage. Indeed in the recent high resolution EM structure of the CPMV eVLP, we were able to resolve the structure of some of this segment for the first time (pink, Fig. 1c)^8^. By contrast, when identical material to that used in cryo-EM was used to grow crystals, the resulting structure did not resolve the C-terminal segment. Subsequent mass spectrometry studies show that over a period of 2–3 weeks, the purified, full length protein undergoes proteolysis of the C-terminal segment^10^, with cleavage being detected at positions 190, 191, and 192 in the S subunit. This difference between eVLP structures determined by cryo-EM and X-ray crystallography could be due to buffer conditions (X-ray crystallography pH 4.7 and cryo-EM pH 7.0), alternative proteolysis, or the different timescales for specimen preparation (days for cryo-EM, weeks for X-ray crystallography). However, given that a 4.4 Å structure of the cleaved particle, that is essentially identical to the X-ray structure, was refined from a subset of the cryo-EM dataset which generated our uncleaved eVLP map, we suggest that timescale is the most important factor (See Supplementary Figure S1).

As well as its inherent interest as a plant pathogen and model system for animal picornaviruses, in recent years CPMV has been extensively used as a tool for biotechnology. A recombinant eVLP has been designed and can easily be produced (>0.5 g/kg of leaf tissue in *N. benthamiana*)^11^. CPMV-M is also of particular interest in biotechnology as it contains a smaller genome segment (RNA-2) than CPMV-B (by 2.5 kb) allowing additional RNA sequences to be inserted into RNA-2 without affecting its ability to be incorporated into particles^12, 13^. Therefore, CPMV-M is an ideal candidate for recombinant virus production for nucleic acid delivery. In addition, as CPMV replication proteins are encoded by RNA-1, preparations of CPMV containing only recombinant RNA-2 are incapable of causing productive infection^14^.

Until now there has been no individual structural analysis of CPMV-T and the RNA packaged into CPMV-M has not been visualised. Here we show high resolution cryo-EM structures of CPMV-M and CPMV-T to 3.9 Å and 4.2 Å respectively. The cryo-EM structures are of sufficient resolution to visualise the individual amino acid side chains and both structures reveal differences between the CPMV particles. Using this information, we have used mutagenesis to confirm the importance of a residue in genome encapsidation and therefore functional viral infection.

## Results and Discussion

### Cryo-EM structure of CPMV-M

**Figure 2:**
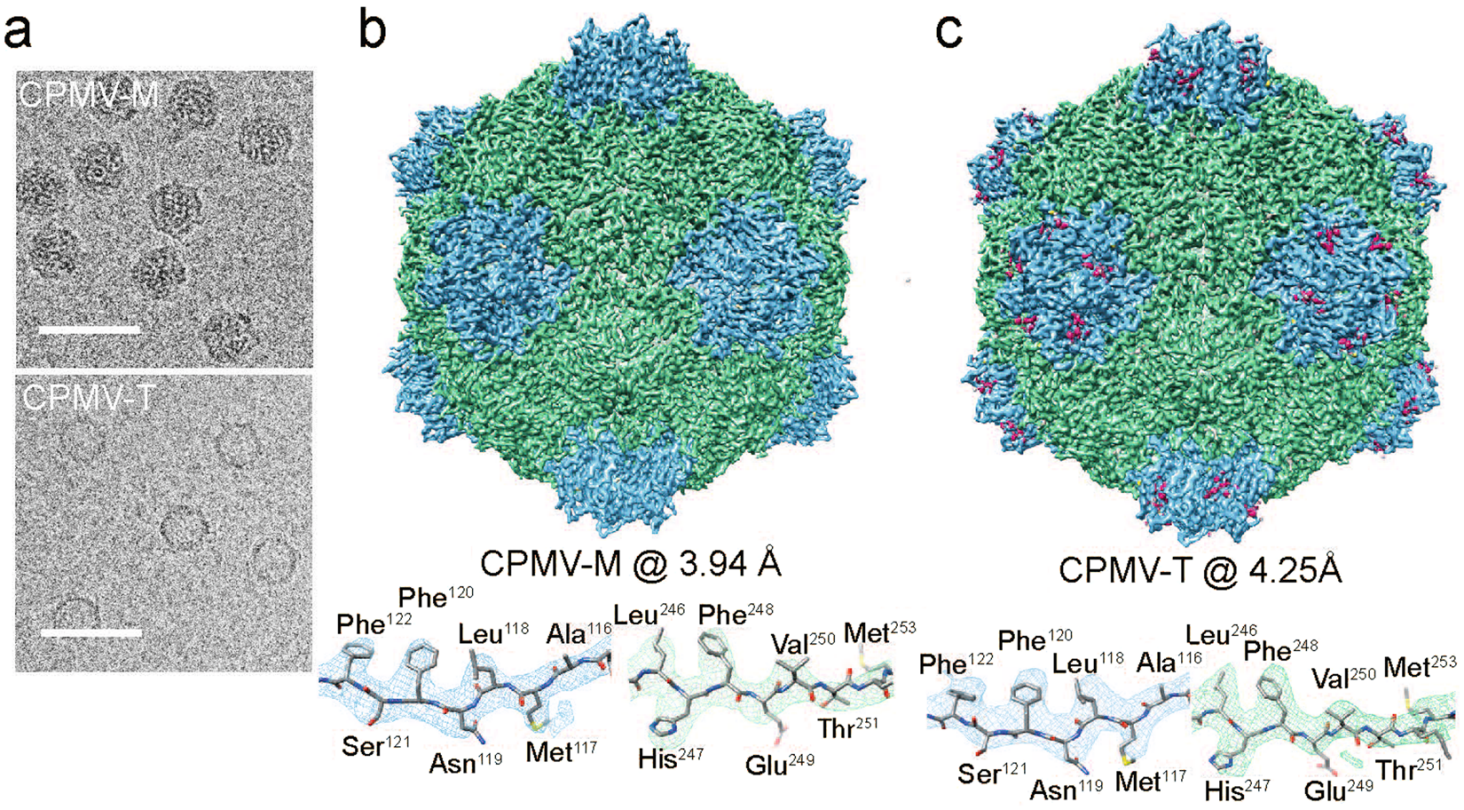
Cryo-EM structure of CPMV-M and CPMV-T. (**a**) Electron micrographs of CPMV-M (top) and CPMV-T (bottom) to show particle distribution. Micrographs were imaged using a Titan Krios electron microscope and detected using a Falcon II direct electron detector (FEI). Scale bar in 50 nm. (**b**) EM density map of CPMV-M (CPMV particle containing RNA-2) determined by cryo-EM to a global resolution of 3.94 Å (EMD-3565). The Large (L) subunit is displayed in green and the Small (S) subunit shown in blue. The bottom panel shows an example of the EM density for an individual β strand from each the L and S subunits displayed as mesh representation. The atomic model within was refined against the EM density. (**c**) EM density map of the naturally occurring empty CPMV particles known as CPMV-T to 4.25 Å (EMD-3562). The view is identical to that shown in (**b**).

Purified CPMV-M was imaged using a direct electron detector on an FEI Titan Krios microscope. A total of 1,759 micrographs of CPMV-M (Fig. 2a, top) were collected. Each micrograph was recorded as an exposure movie containing seven images which were corrected for drift and beam-induced movement^15^. EM processing was carried out in RELION (v1.3)^16^ (see experimental methods for details). A total of 24,976 particles were automatically picked^16^ from the CPMV-M dataset. Classification (both 2D and 3D) was used to select a homogenous subset of particles. The homogenous subset (10,850 particles) was used for 3D refinement. The resulting density map was sharpened using an empirically-derived B-factor of −144.1 Å^2^, to 3.94 Å (Fig. 2b; EMD-3565).

**Figure 3:**
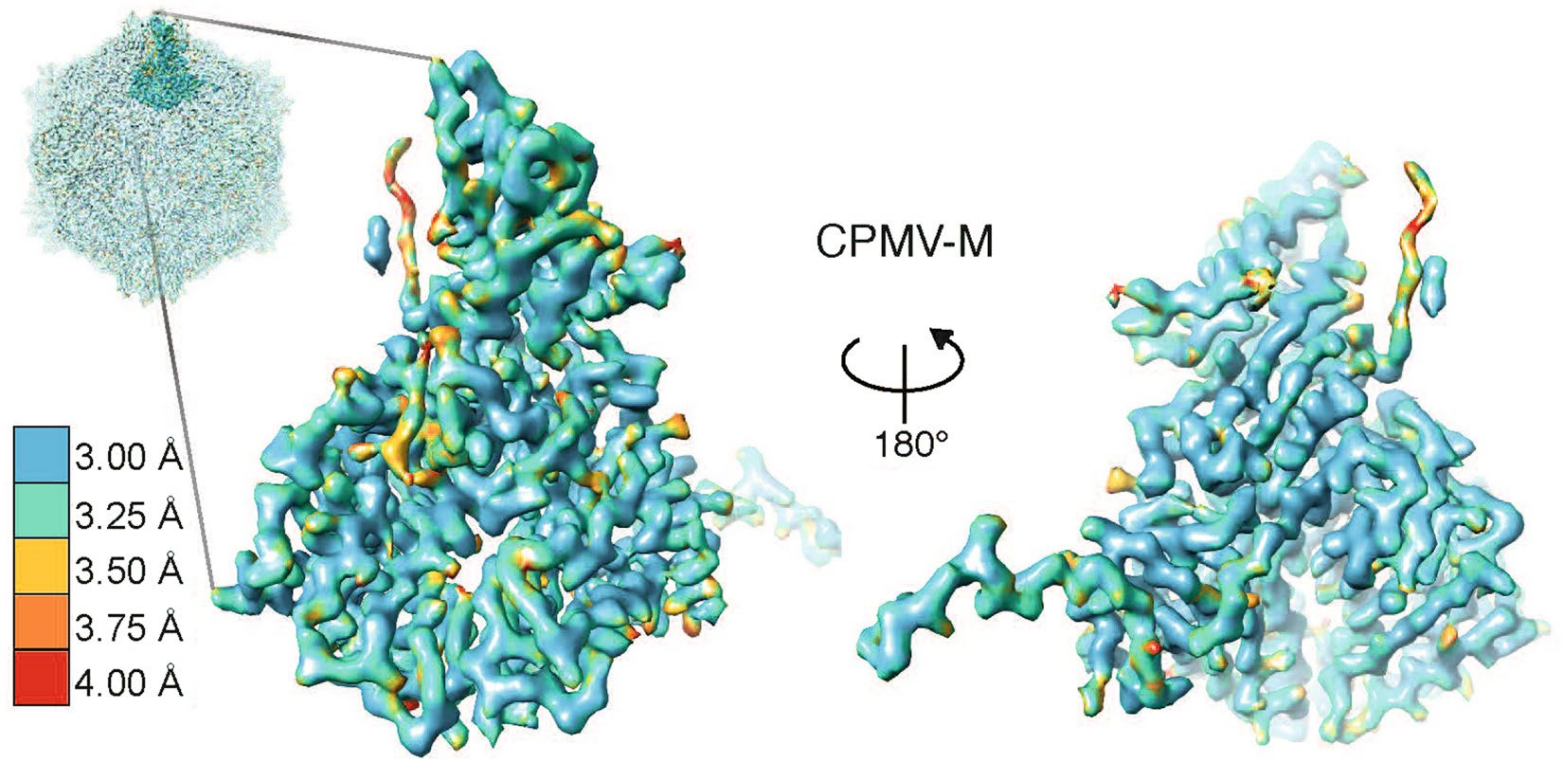
Local resolution of CPMV-M. A single asymmetric unit of CPMV-M (seen from outside the capsid) is shown coloured according to its local resolution. The interior of the asymmetric unit is also shown by rotating the asymmetric unit 180° around the y-axis. The highest resolution bin is 3.00 Å (blue) and the lowest resolution bin is 4.00 Å (red). A key is shown for reference.

The resolution of the map permits the side chains of the large amino acids to be visualised (Fig. 2b). To analyse the individual amino acids, the atomic model for CPMV-B, derived from cryo-EM density (PDB 5a32), was docked into the CPMV-M EM map using Chimera^17^. Coot^18^ was used to analyse each individual residue. EM density for S subunit residues 184 to 189 inclusive were not visible; these residues were removed from the model. The resulting model was fitted into the density using the Rosetta ‘relax’ protocol^19^ to give the model presented in Fig. 2b. The quality of the model was assessed using Molprobity^20^ and the statistics suggest this model is in the 97^th^ percentile of atomic models available (Table 1). The overall resolution of CPMV-M is 3.9 Å; however, several regions of the EM density, notably for the β strands shown in Fig. 2b, appear to be at significantly higher resolution. To analyse this region-specific resolution in the CPMV-M map, the local resolution was determined using ResMap (Fig. 3)^21^. Using this method, the majority of the map is shown to be between 3.0 and 3.25 Å. A single asymmetric unit of CPMV-M (Fig. 3) shows low resolution information at the C terminus of S subunit (red/orange) suggesting this region of the capsid may be flexible. Other low resolution areas appear to be in exterior loops of the capsid. Rotation of 180° around the y-axis reveals the region of the asymmetric unit which is on the interior of the capsid (Fig. 3, bottom). Here, the majority of the EM density is at 3.0 Å resolution, suggesting less flexibility, presumably as the interior of the capsid is a more stable environment.

**Table 1:** Microscopy and image processing details for CPMV-M and CPMV-T.

	CPMV-M	CPMV-T
**Data Collection**		
Particles in final reconstruction	10,850	4,696
Pixel size (Å/pixel)	1.10	1.10
Defocus range (μm)	0.35–10.0	0.35–8.0
Voltage (kV)	300	300
Dose (e^−^/Å^2^)	42	42
**Particle Numbers**		
Autopicking	24,976	5,594
Particle sorting	21,931	5,457
2D classification	15,228	5,165
3D classification	10,850	4,696
**EM Refinement**		
Final resolution (Å)	3.94	4.25
Experimental B-factor (Å^2^)	−144.1	−185.8
EMDB accession number	EMD-3565	EMD-3562
**Model Validation**		
MolProbity score	1.41 (97^th^ percentile)	1.32 (98^th^ percentile)
Clashscore (all atoms)	1.77 (99^th^ percentile)	1.51 (99^th^ percentile)
Ramachandran favoured	92.34%	93.45%
PDB ID	5MSH	5MS1

### Organisation of RNA-2 within the CPMV-M capsid

**Figure 4:**
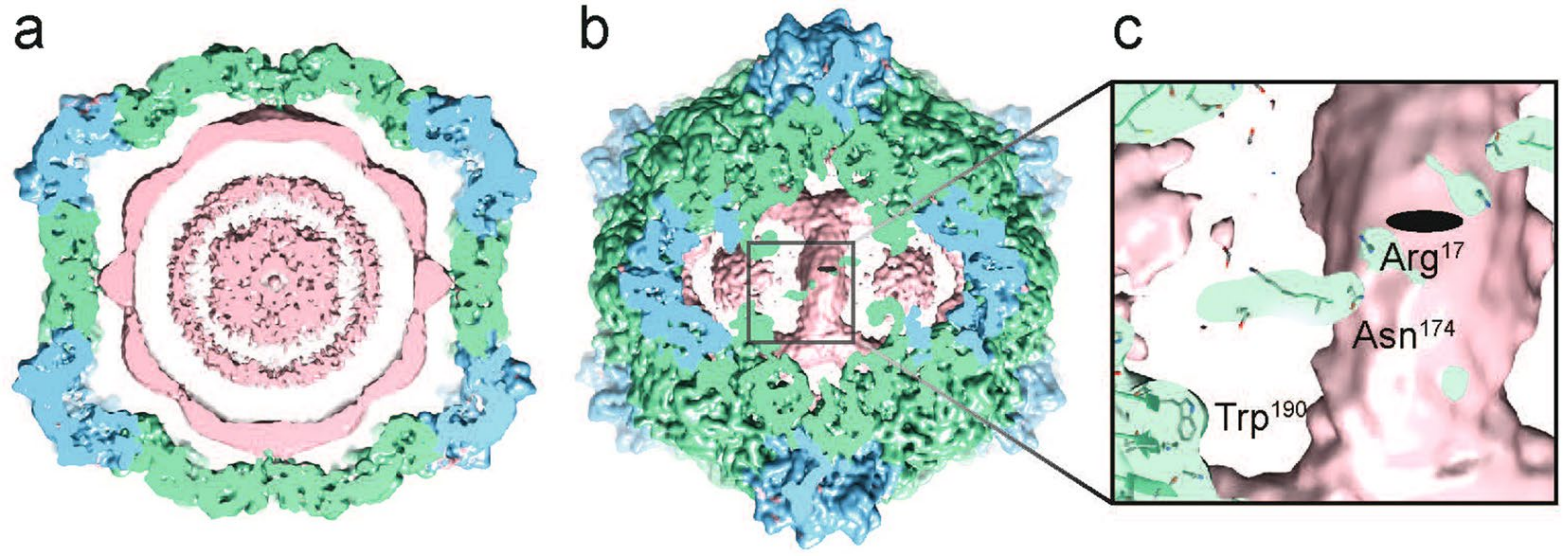
Genome organisation of CPMV-M containing RNA-2. (**a**) A 40 Å thick central slab through the unsharpened CPMV-M EM map (at 4.25 Å resolution; also deposited with the EMD-3565 deposition. Suggested contour level is 0.012). A view down the two-fold axis is shown. The coat proteins are coloured as before with the extra EM density attributed to RNA coloured pink. (**b**) A view beneath the capsid at the two-fold axis showing the strongest density for RNA. (**c**) Zoomed in view to highlight RNA-protein interactions. The strongest density bridges between the capsid and RNA are at amino acids Asn174 and Arg17 from the L subunit. Trp190 binds strongly to RNA in CPMV-B virions (containing RNA-1), here we can see no density between Trp190 and the EM density attributed to RNA-2.

CPMV-M particles contain RNA-2, a single stranded RNA molecule 3.5 kb in length. EM density is visible within the CPMV-M capsid that can be attributed to RNA-2 (pink, Fig. 4). The RNA density is only visible in the unsharpened CPMV-M map (4.25 Å, the unsharpened map is deposited alongside EMD-3565). B-factor sharpening is used to improve high resolution features such as amino acid side chains, and simultaneously removes low resolution information such as the relatively poorly ordered RNA-2 in CPMV-M. RNA-2 is 2.6 kb smaller than RNA-1 and in this CPMV-M cryo-EM structure, less density is visualised within the capsid than seen previously for CPMV-B at equivalent contour levels^8^. The RNA-2 molecule appears as concentric shells of density (Fig. 4a), as seen for other icosahedral viruses^8, 22–24^. The outside shell is dodecahedral, reflecting the icosahedrally averaged position of the RNA in the capsid, and so the exact structure of the presumably asymmetric RNA-2 cannot be deduced. However, clear interactions between the protein capsid shell can be seen beneath the two-fold axis (Fig. 4b). This is exactly where RNA-1 in CPMV-B structure interacts with the capsid^8^. This is not the same for all comoviruses; a crystal structure of BPMV shows RNA-capsid binding at the three-fold axis^3, 4^.

**Figure 5:**
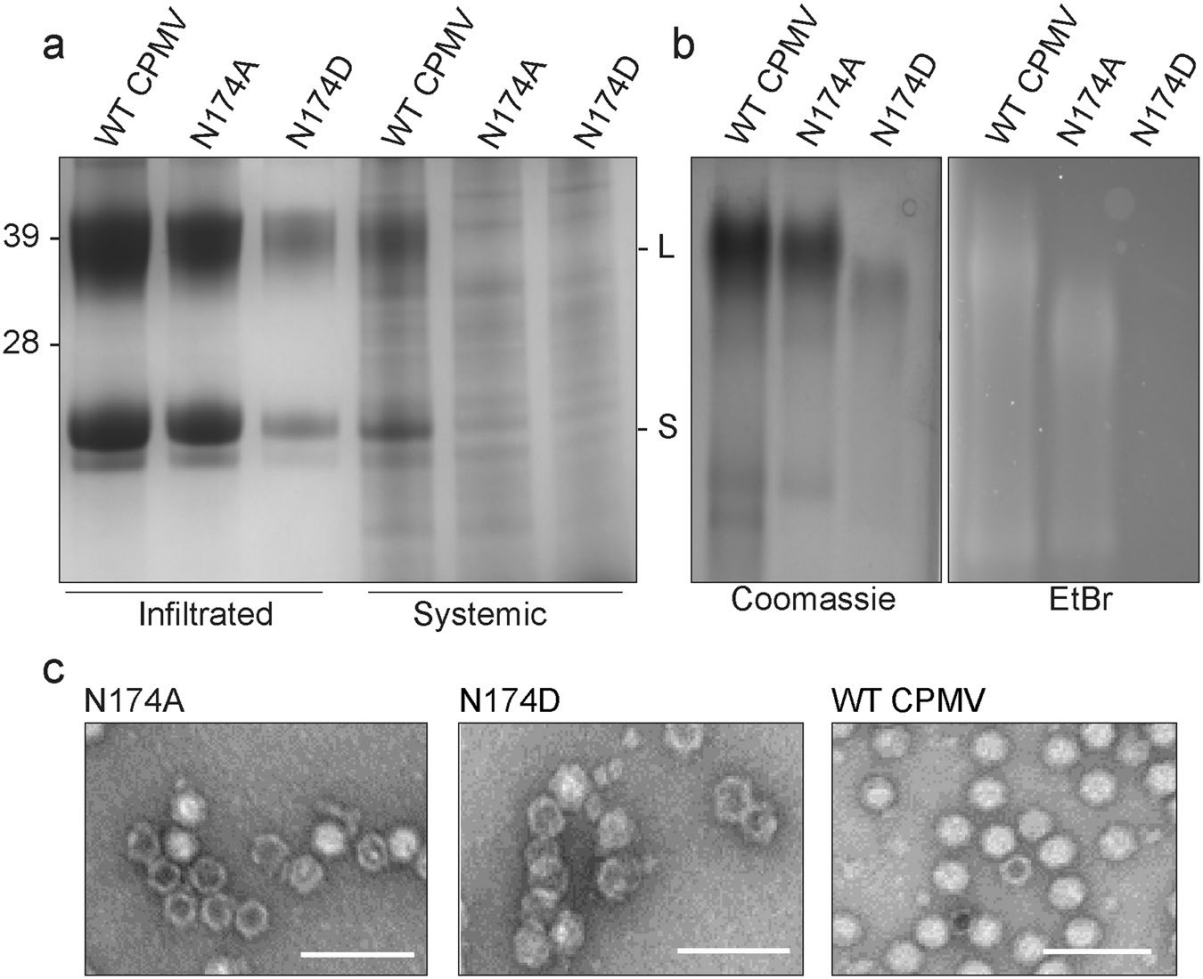
Asn174 in the Large subunit is essential for a systemic infection. (**a**) For each mutant and WT virion 15 grams of leaves were analysed. Coomassie blue stained SDS PAGE shows the mutation N174A results in similar levels of the Large (L) and small (S) coat proteins to those found with WT virus being produced in infiltrated leaves. By contrast the N174D mutation resulted in a substantial reduction in the amounts of the two coat proteins. Both mutations inhibit CPMV from causing a systemic infection, with the L and S coat proteins being undetectable in extracts. (**b**) Coomassie blue and ethidium bromide (EtBr) stained native agarose gels of particles isolated from infiltrated leaves. The N174A mutation allows genome encapsidation while N174D mutant does not appear to package RNA. (**c**) Negative stain EM shows N174A and N174D mutants appear to assemble as complete particles similar to WT CPMV (also shown for reference). Scale bar is 100 µm.

Discrete bridges of density between the RNA and the protein capsid are observed (Fig. 4c), which appear to correspond to interactions between two amino acids from the L subunit, Asn174 and Arg17, and RNA-2 (Fig. 4c). A cryo-EM reconstruction of CPMV-B also identified Arg17 as an amino acid essential for RNA encapsidation^8^, however a prominent interaction between Asn174 and the genomic RNA was not identified. To ascertain if Asn174 is essential for RNA packaging it was mutated to alanine (A) and aspartic acid (D) in an infectious clone of RNA-2 (Fig. 5). When the modified RNA-2 constructs were agroinfiltrated into *Nicotiana benthamiana* in the presence of RNA-1, it was found that the N174A mutant behaved similarly to WT CPMV in infiltrated leaves in terms of the production of virus particles (Fig. 5). However, N174A was unable to cause a systemic infection, as judged by the lack of symptoms on the upper leaves and the lack of any viral particles in extracts prepared from them. Despite this, N174A mutant particles, produced in the infiltrated leaves, appeared to encapsidate RNA (Fig. 5b). Both mutants were able to form particles in infiltrated leaves apparently identical to WT CPMV as judged by electron microscopy (Fig. 5c). Mutation of N174D resulted in a more pronounced effect, the viral yield from infiltrated leaves was markedly reduced, no systemic infection was observed and RNA was not encapsidated (Fig. 5). Due to the proximity of N174 and R17 (shown previously to be critical for RNA encapsidation^8^) it is possible the introduction of N174D may interfere with R17 by formation of a salt bridge which could explain the more severe effect of N174D compared with N174A. Reduced RNA encapsidation has previously been shown to adversely affect CPMV yield and systemic spread^6^, thus the phenotypes of these mutants are consistent with reduced RNA encapsidation. In addition, mutations of N174 in eVLP (to A or D) do not affect capsid assembly (not shown), showing these mutations do not substantially change the protein:protein interactions which hold the capsid together. Together these data suggest Asn174 is important for genome encapsidation. The cryo-EM reconstruction of CPMV-B shows a large bridge of density between encapsidated RNA-1 and a tryptophan residue (W190) in the L subunit of the capsid^8^; by contrast, no interaction between RNA and W190 can be visualised in the CPMV-M structure (Fig. 4c).

### Cryo-EM structure of CPMV-T

CPMV-T was imaged and analysed in the same way as CPMV-M (above). A total of 1,324 micrographs of CPMV-T (Fig. 2a, bottom) were acquired. The micrographs were automatically picked^16^ generating a total dataset of 5,594 particles, which were classified (by both 2D and 3D) to select a homogenous subset of particles (please see experimental methods for details). The homogenous subset (4,696 for CPMV-T) was used for 3D refinement. The refined CPMV-T EM density map was sharpened using empirically-derived B-factor of −185.8 Å^2^ to 4.25 Å resolution (Fig. 2c; EMD-3562).

**Figure 6:**
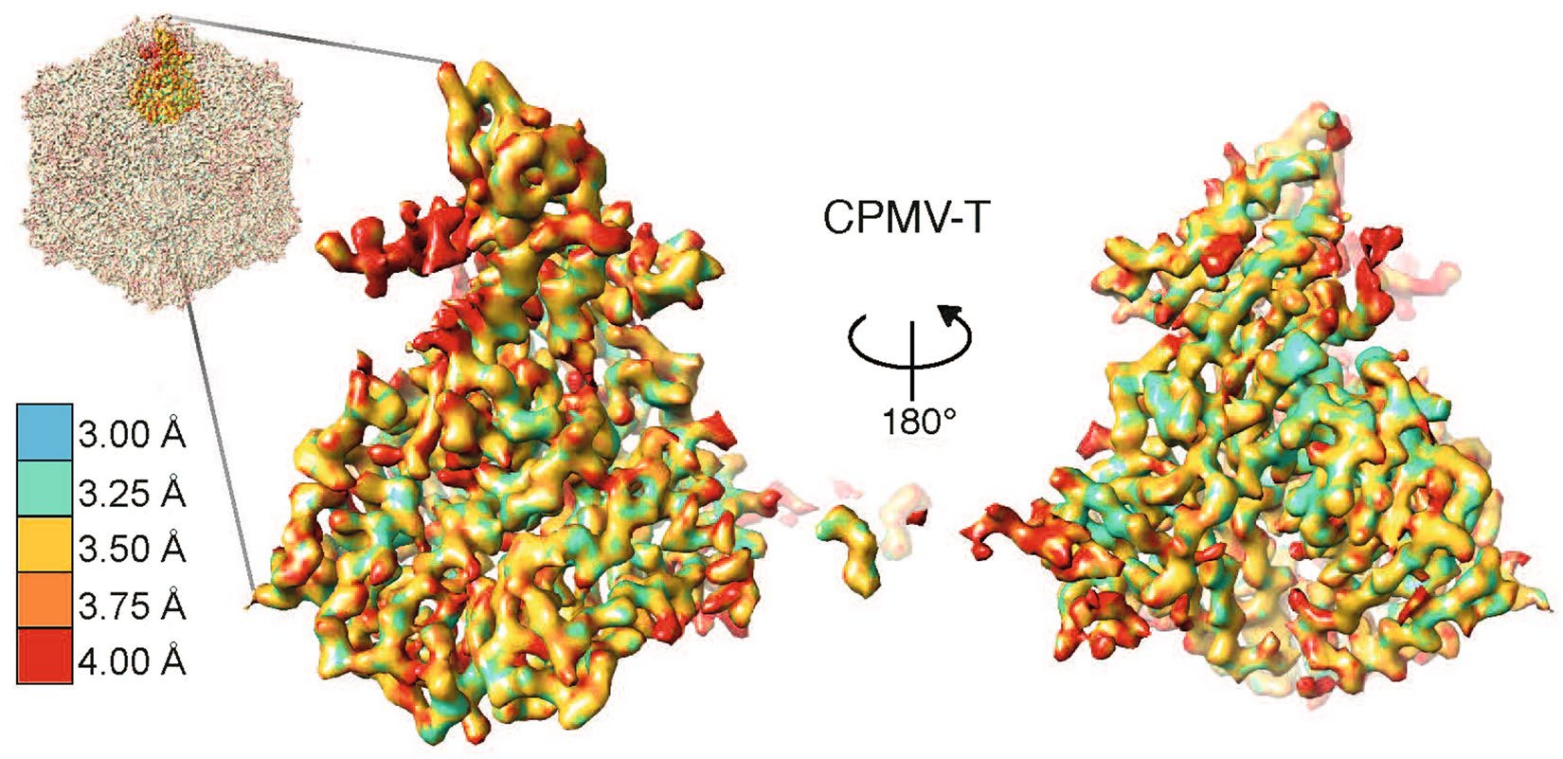
Local resolution of CPMV-T. A single asymmetric unit of CPMV-T is shown coloured according to its local resolution (the views and colour scheme are identical to those in Fig. 3). The interior of the asymmetric unit is also shown by rotating it 180° along the y-axis. The highest resolution bin is 3.00 Å (blue) and the lowest resolution bin is 4.00 Å (red). A key is shown for reference.

The resolution of the map was again high enough for the side chains of the large amino acids to be identified (Fig. 2c). To analyse the individual amino acids within the cryo-EM structure, the CPMV eVLP atomic model (PDB: 5a33) was docked into the EM density using Chimera^17^. This atomic model was used because a simple examination of the EM density map revealed that extra C-terminal density, similar to that in the CPMV eVLP EM derived model was present. Coot^18^ was used to remove residues 1 to 7 and 189 to 202 from the S subunit where no density was visible in the CPMV-T map. New regions of density in the C terminus of the S subunit were visible and the position of three amino acids (residues 184, 188 and 189) were built into the model using Coot^18^. The resulting model was fitted into the cryo-EM density using the Rosetta ‘relax’ protocol^19^, generating the model presented in Fig. 2. The EM density for the β strands shown in Fig. 2c shows defined density for the side chains which appears to be at a higher resolution than 4.25 Å. The local resolution of CPMV-T^21^ confirms this, and shows that much of the map is at ~3.5 Å (Fig. 6). The C terminus of the S subunit (red) is the lowest resolution part of the map (>4 Å) suggesting flexibility in this region. Many parts in the interior of the capsid show resolution that extends to ~3.25 Å and the statistics (generated using MolProbity) suggest this model is in the 98^th^ percentile of atomic models available (Table 1).

### The structure of the S subunit C terminal extension

**Figure 7:**
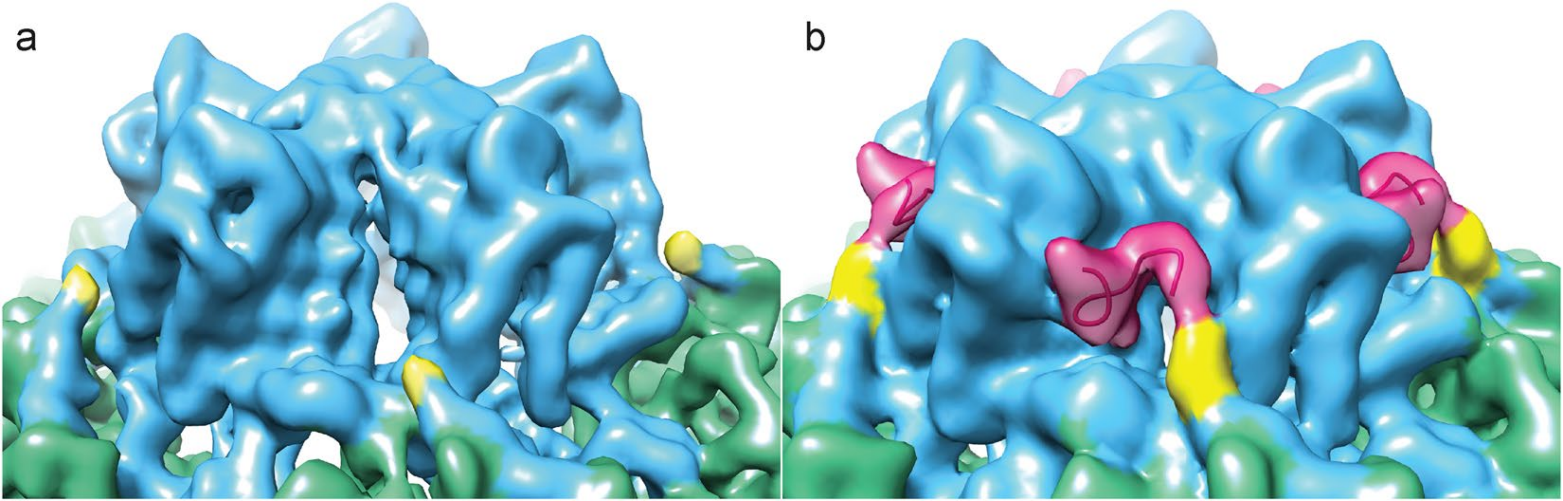
The C terminus extension of S subunit. (**a**) The unsharpened EM density map of CPMV-M. The S subunit is coloured blue and the L subunit is in green. The C terminal amino acid in the CPMV-M map is Ser183 and is coloured yellow. (**b**) The unsharpened EM density map of CPMV-T. Coloured as in (**a**). Amino acids Ser183 and Thr184 are coloured yellow. The C terminus extension, only visible in the CPMV-T map, amino acids Pro188 to Ile197 are coloured magenta.

The structure of the majority of the CPMV-M and CPMV-T capsid is essentially identical, but the C terminal segment of the S subunit shows a number of differences (Fig. 7). The C terminal 24 amino acids of the S subunit are cleaved following capsid formation and genome encapsidation^7^. Current structural studies of WT CPMV show the final residue as Lys189, and so the last 24 amino acids are missing due to this cleavage^1, 8^. The final C terminal residue built into the CPMV-M map is Ser183 (Fig. 7a), which is six amino acids shorter than in the CPMV-B structures published previously^1, 8^. Mass spectrometry and proteomics analysis of WT CPMV (containing a mixture of CPMV-B, CPMV-M and CPMV-T) identified a number of different C termini in the S subunit fast electrophoretic band; however, cleavage between Ser183 and Thr184 was not identified^10^. We therefore suggest that residues 184–189 are present in the particle, but disordered and missing from the density map. The absence (or lack of defined structure) of these amino acids potentially leaves a gap in the capsid shell and so the inside of the capsid is accessible to the exterior environment. Analysis of Brome Mosaic Virus (BMV), a virus with three genomic RNA segments, which are packaged separately, has shown that the different genomic RNAs are released at different times, helping to regulate the timing of gene expression^25^. The different structures of CPMV-B and CPMV-M could provide a structural basis for such effects, with the CPMV-M capsid appearing to be less stable than the CPMV-B capsid. However, further studies will be required to validate this idea, and identify the routes for allosteric communication that allow an effect on capsid structure to be driven by different genomic RNA binding on the inside surface of the capsid.

In the CPMV-T map, EM density up to residue Thr184 can be visualised, after which the density becomes disordered and the next residue that can be convincingly built into the CPMV-T map is Pro188. The final visible residue is Ile197 (Fig. 7b). Thus, although the structure of the C terminus of the S subunit in our CPMV-T map (the naturally occurring empty particles) has some differences to the previously determined CPMV eVLP (recombinant empty particles), broadly the structures of the two are similar. They both contain a disordered region where there is no high-resolution density. This is residues 184–189 in the eVLP and residues 185–187 in the CPMV-T structure presented here. Previous proteomic studies have not identified any proteolysis sites before residue 190, suggesting that proteolysis in this region is unlikely to account for the lack of high-resolution density for this segment. Furthermore, although there is no high-resolution density, in the unsharpened map there is continuous density that encompasses the path of the polypeptide chain that bridges the gap (See ref. 8 and Fig. 7). This strongly suggests that this segment of polypeptide is present, uncleaved but relatively poorly ordered in both the eVLP and CPMV-T cryo-EM structures. The differences between CPMV-T and eVLP are likely a consequence of the amount and quality of data in the two reconstructions, leading to a lower resolution for CPMV-T than for eVLP (4.2 Å vs 3.0 Å for the eVLP). The different conformation of the C-terminus (extending to Arg193) seen in the X-ray structure of the eVLP^10^ is consistent with the conformation seen in the CPMV-B^1, 8^ and the CPMV-M map presented here, confirming previous proteomic studies that show the S-subunit has been cleaved^6, 10^. In the cryo-EM structure of eVLP a number of important amino acids were identified in the C terminus extension. Mutational analysis of these amino acids (F192, F194, V109, R193 and E147 of the S subunit) demonstrated they were involved in genome encapsidation and/or capsid assembly and the role of the C terminal extension is to stabilise the interactions between the S subunits at the pentamer^8^. The same interactions are also visible in the CPMV-T map presented in this study^8^.

Previous work shows that the C-terminus of the S subunit is proteolytically sensitive in all CPMV particles, but more so in those that contain genomic RNA^6, 8, 10, 26^. By contrast, both the naturally occurring CPMV-T and the engineered eVLP^8^ which lack any RNA are cleaved more slowly, and thus retain the C-terminus for longer. Cleavage of the C terminal peptide reveals a large hydrophobic patch on the surface of CPMV. The presence of an intact C-terminal sequence appears to be important for efficient intercellular movement of the virus, since genetic removal of this region results in impaired movement of the virus within the plant^6^, a phenomenon associated with virus aggregation within cells (Meshcheriakova, Y and Lomonossoff, G.P., unpublished). Thus, the C-terminal sequence may have a role in occluding this hydrophobic patch thereby preventing aggregation and enhancing viral movement, rather than facilitating direct interactions between the C-terminus and the viral movement protein.

In this study, we have produced the first near atomic resolution structures of CPMV-M and CPMV-T. These structures have permitted us to identify key differences between the forms of CPMV. The CPMV structures are similar, particularly in the L subunit. The main differences are between the empty CPMV particles (CPMV-T and eVLP) and CPMV particles containing RNA (CPMV-B and CPMV-M). We have demonstrated the importance of specific amino acids in genome encapsidation. Of particular interest is N174 in the L subunit, which was identified by its interaction with density attributed to RNA. Mutational analysis demonstrated N174 is involved in RNA encapsidation but not particle assembly. In the CPMV-B^8^ structure (which encapsidates RNA-1) the most prominent RNA:protein interaction is between W190 of the L subunit. Intriguingly, density linking W190 to the RNA density is not visible in the structure of CPMV-M (containing RNA-2) reported here. CPMV-M cannot replicate alone in host cells as it does not contain sequences encoding the replication proteins. CPMV-M is therefore an ideal candidate for drug or nucleic acid delivery^12, 27^. RNA-2 has been modified for use in diagnostic RT-PCR^14^ and more recently, it has been modified to remove the region encoding the viral movement protein, thereby abolishing the ability of the virus to move from cell to cell^27, 28^. This study provides the first structural analysis of purified CPMV-M particles. This information should aid the further development of CPMV-M as a particle for the encapsidation of specific RNA molecules for diagnostic or therapeutic purposes.

## Methods

### CPMV-M and CPMV-T purification

Infection of *N. benthamiana* with CPMV was initiated by agroinfiltration of plants with pBinP-S1NT and pBinP-S2NT^29^ and the resulting virus particles were purified^30^. The individual components were separated by centrifugation on 42%, 49%, 57% and 65% (w/v) CsCl gradients^31^.

### Mutagenesis

Point mutations were introduced into the coat protein-coding region of either pEAQ-RNA-2, a plasmid containing a full-length copy of CPMV RNA-2 in pEAQ-*HT* using the GENEART® Site-Directed Mutagenesis System (Invitrogen™) according to manufacturer's protocol. Primers for site-directed mutagenesis were designed using QuikChange Primer Design Program. To examine the effect of the mutations on virus infectivity and RNA encapsidation, the mutant forms of pEAQ-RNA-2 were co-infiltrated into *N. benthamiana* with pBinP-S1NT and any particles produced were purified as described for WT CPMV (above).

### Grid preparation and imaging

Cryo-EM grids were prepared by placing 3 μl of ~7.9 mg/ml CPMV-M or ~1.92 mg/ml CPMV-T onto 200 mesh grids with 2 μm holes (Quantifoil R2/2, Quantifoil Micro Tools, GmbH, Germany). Grids were glow discharged for ~20 seconds prior to plunge freezing in liquid ethane cooled by liquid nitrogen, using a FEI Vitrobot IV at 100% relative humidity, chamber temperature of 4 °C. Data was collected on an FEI Titan Krios (NeCEN, Leiden, The Netherlands) transmission electron microscope at 300 kV, using an electron dose of 42 e^−^/Å^2^ and a magnification of 125,085x. The final object sampling was therefore of 1.10 Å/pixel. A total of 1,323 (CPMV-T) and 1,759 (CPMV-M) exposures were recorded using the EPU automated acquisition software on a 17 Hz FEI Falcon II direct electron detector. Each exposure movie had a total exposure of one seconds and contained seven images.

### Image processing

Drift-corrected averages of each movie were created using MOTIONCORR^15^ and the contrast transfer function of each determined using CTFFIND3^32^ any images showing signs of significant astigmatism were discarded. All subsequent image processing steps were performed using RELION (v1.3)^16, 33^ unless otherwise stated. Approximately 1,000 particles were manually picked and classified using reference-free 2D classification. The resulting 2D class average views were used as templates for automated particle picking^16^ (see Table 1 for particle numbers at each processing step). Particles were sorted using a statistical sorting algorithm based on how similar particles are to the reference images^16^ and the ‘worst’ 10% of particles were discarded. The remaining particles were classified using several rounds of both reference free 2D classification and 3D classification, with icosahedral (I3) symmetry imposed. For both CPMV-M and CPMV-T, the initial starting model was the CPMV eVLP structure (EMD-3014^8^) filtered to ~60 Å resolution, a resolution at which almost all information other than size, shape and symmetry of the structure are removed. After each round, the best classes/class was taken to the next step of classification. To correct for mechanical drift, beam-induced movement and radiation damage, statistical movie processing and particle polishing procedures were implemented^34^. As CPMV particles are readily visible even in individual movie frames, a running average of three frames was used in the calculations. Post-processing was employed to appropriately mask the model, estimate and correct for the B-factor of the maps^35^. The final resolution was determined using the ‘gold standard’ Fourier shell correlation (FSC = 0.143) criterion^33^ 4.25 Å for CPMV-T and 3.94 Å for CPMV-M. Local resolution was estimated using the ResMap wrapper in RELION^21^.

### Refinement of atomic models

During model refinement, the EM density map was fixed and the atomic model was refined against that fixed map. The EM derived atomic model of CPMV-B (PDB: 5a32^8^) was fitted into the CPMV-M EM map using Chimera as a simple examination revealed that no density for the C-terminus was present. Residues present in the CPMV-B model but not in the CPMV-M EM map (residues 184 to 189 of the S subunit) were deleted using Coot^18^ and the resulting model was refined using the ‘relax’ protocol in Rosetta^19^. The eVLP CPMV EM derived atomic model (PDB: 5a33^8^) was fitted in to CPMV-T EM map because a simple examination of the EM density map revealed that extra C-terminal density, similar to that in the CPMV eVLP EM derived model was present. Residues in the CPMV eVLP model not present in CPMV-T map were deleted (residues 1 to 5 and 198 to 202 of the S subunit) using Coot^18^. Amino acids that were previously not visualised (residues 184 and 188–189) were added and modeled in Coot^18^. The model was subsequently refined using the ‘relax’ protocol in Rosetta^19^ and assessed for quality using MolProbity (Table 1). Figures were generated using Chimera^17^.

## Electronic supplementary material

Supplementary Information

## References

[1] Lin T (1999). The refined crystal structure of cowpea mosaic virus at 2.8 A resolution. Virology.

[2] Ochoa WF, Chatterji A, Lin T, Johnson JE (2006). Generation and structural analysis of reactive empty particles derived from an icosahedral virus. Chem Biol.

[3] Lin T, Cavarelli J, Johnson JE (2003). Evidence for assembly-dependent folding of protein and RNA in an icosahedral virus. Virology.

[4] Chen ZG (1989). Protein-RNA interactions in an icosahedral virus at 3.0 A resolution. Science.

[5] Lin T (2000). Structural fingerprinting: subgrouping of comoviruses by structural studies of red clover mottle virus to 2.4-A resolution and comparisons with other comoviruses. J Virol.

[6] Taylor KM, Spall VE, Butler PJ, Lomonossoff GP (1999). The cleavable carboxyl-terminus of the small coat protein of cowpea mosaic virus is involved in RNA encapsidation. Virology.

[7] Lomonossoff GP, Johnson JE (1991). The synthesis and structure of comovirus capsids. Prog Biophys Mol Biol.

[8] Hesketh EL (2015). Mechanisms of assembly and genome packaging in an RNA virus revealed by high-resolution cryo-EM. Nature Communications.

[9] Saunders K, Sainsbury F, Lomonossoff GP (2009). Efficient generation of cowpea mosaic virus empty virus-like particles by the proteolytic processing of precursors in insect cells and plants. Virology.

[10] Huynh NT (2016). Crystal Structure and Proteomics Analysis of Empty Virus-like Particles of Cowpea Mosaic Virus. Structure.

[11] Sainsbury F, Thuenemann EC, Lomonossoff GP (2009). pEAQ: versatile expression vectors for easy and quick transient expression of heterologous proteins in plants. Plant Biotechnol J.

[12] Gopinath K (2000). Engineering Cowpea Mosaic Virus RNA-2 into a Vector to Express Heterologous Proteins in Plants. Virology.

[13] Monger W (2006). An antibody derivative expressed from viral vectors passively immunizes pigs against transmissible gastroenteritis virus infection when supplied orally in crude plant extracts. Plant Biotechnol J.

[14] King DP, Montague N, Ebert K, Reid SM (2007). Development of a novel recombinant encapsidated RNA particle: evaluation as an internal control for diagnostic RT-PCR. J Virol Methods.

[15] Li X (2013). Electron counting and beam-induced motion correction enable near-atomic-resolution single-particle cryo-EM. Nat Methods.

[16] Scheres SHW (2015). Semi-automated selection of cryo-EM particles in RELION-1.3. J Struct Biol.

[17] Pettersen EF (2004). UCSF Chimera–a visualization system for exploratory research and analysis. J Comput Chem.

[18] Emsley P, Lohkamp B, Scott WG, Cowtan K (2010). Features and development of Coot. Acta Crystallogr D Biol Crystallogr.

[19] DiMaio F (2015). Atomic-accuracy models from 4.5-A cryo-electron microscopy data with density-guided iterative local refinement. Nat Methods.

[20] Chen VB (2010). MolProbity: all-atom structure validation for macromolecular crystallography. Acta Crystallogr D Biol Crystallogr.

[21] Kucukelbir A, Sigworth FJ, Tagare HD (2014). Quantifying the local resolution of cryo-EM density maps. Nat Methods.

[22] Bakker SE (2014). Limits of structural plasticity in a picornavirus capsid revealed by a massively expanded equine rhinitis A virus particle. J Virol.

[23] Toropova K, Basnak G, Twarock R, Stockley PG, Ranson NA (2008). The three-dimensional structure of genomic RNA in bacteriophage MS2: implications for assembly. J Mol Biol.

[24] Hurdiss DL (2016). New Structural Insights into the Genome and Minor Capsid Proteins of BK Polyomavirus using Cryo-Electron Microscopy. Structure.

[25] Vaughan R (2014). The Tripartite Virions of the Brome Mosaic Virus Have Distinct Physical Properties That Affect the Timing of the Infection Process. J Virol.

[26] Sainsbury F, Saunders K, Aljabali AAA, Evans DJ, Lomonossoff GP (2011). Peptide-controlled access to the interior surface of empty virus nanoparticles. Chembiochem.

[27] Madi M, Mioulet V, King DP, Lomonossoff GP (2015). & Montague, N. P. Development of a non-infectious encapsidated positive control RNA for molecular assays to detect foot-and-mouth disease virus. J Virol Methods.

[28] Wellink J, van Kammen A (1989). Cell-to-cell Transport of Cowpea Mosaic Virus Requires Both the 58K/48K Proteins and the Capsid Proteins. J Gen Virol.

[29] Liu L, Lomonossoff G (2002). Agroinfection as a rapid method for propagating Cowpea mosaic virus-based constructs. J Virol Methods.

[30] van Kammen A (1967). Purification and properties of the components of cowpea mosaic virus. Virology.

[31] Holness CL, Lomonossoff GP, Evans D, Maule AJ (1989). Identification of the initiation codons for translation of cowpea mosaic virus middle component RNA using site-directed mutagenesis of an infectious cDNA clone. Virology.

[32] Mindell JA, Grigorieff N (2003). Accurate determination of local defocus and specimen tilt in electron microscopy. J Struct Biol.

[33] Scheres SHW, Chen S (2012). Prevention of overfitting in cryo-EM structure determination. Nat Methods.

[34] Scheres SHW (2012). RELION: implementation of a Bayesian approach to cryo-EM structure determination. J Struct Biol.

[35] Chen S (2013). High-resolution noise substitution to measure overfitting and validate resolution in 3D structure determination by single particle electron cryomicroscopy. Ultramicroscopy.

