# Supplementary material for: The structures of a naturally empty cowpea mosaic virus particle and its genome-containing counterpart by cryo-electron microscopy
Source: Sci Rep. 2017 Apr 3;7:539. doi: 10.1038/s41598-017-00533-w (PMC5428714; doi:10.1038/s41598-017-00533-w)
Supplement: Supplementary file 1 — Supplementary Information [file 41598_2017_533_MOESM1_ESM.pdf]

Supplementary Information

The structures of a naturally empty cowpea mosaic virus particle and its genome-containing counterpart by cryo-electron microscopy

Emma L. Hesketh[1†], Yulia Meshcheriakova[2], Rebecca F. Thompson[1], George P. Lomonossoff[2] & Neil A. Ranson[1†].

[1] Astbury Centre for Structural Molecular Biology, University of Leeds, Leeds, LS2 9JT. UK.
[2] Dept. of Biological Chemistry, John Innes Centre, Norwich Research Park, Colney, Norwich NR4 7UH, UK

[†] Co-corresponding authors: e.l.hesketh@leeds.ac.uk, n.a.ranson@leeds.ac.uk

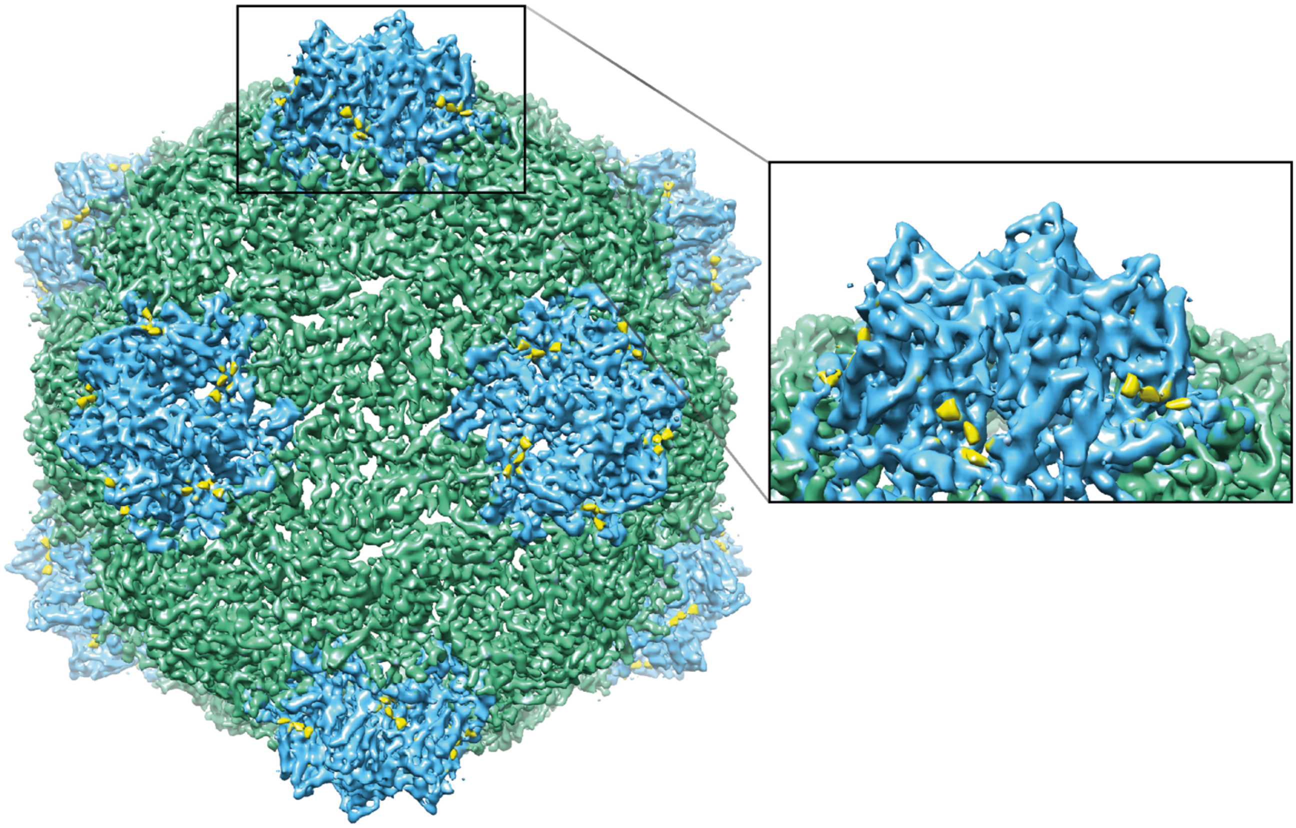

Figure S1. ***Cleaved*** **4.4 Å eVLP structure (EMD-8609).**

This *cleaved* eVLP structure was refined from a small subset of data from the cryo-EM reconstruction of *uncleaved* eVLP reported previously (Hesketh et al., 2015). The Large Subunit is coloured green and the Small Subunit is coloured blue. The structure of CPMV-B (PDB:5a32) is fitted into this map. Residues 183 – 189 in the Small Subunit are coloured yellow. The map was reconstructed from 1,537 particles in Relion1.3. All microscopy and pre-processing was performed as described previously (Hesketh et al., 2015). The density map was sharpened using an empirically derived B-factor of -156.7 Å^2 to 4.4 Å resolution.

Hesketh, E. L. *et al.* Mechanisms of assembly and genome packaging in an RNA virus revealed by high-resolution cryo-EM. *Nature Communications* **6,** 10113 (2015).
